# Pediatric nurses in pediatricians’ offices: a survey for primary care pediatricians

**DOI:** 10.1186/s12875-021-01457-1

**Published:** 2021-06-29

**Authors:** Immacolata Dall’Oglio, Giovanni Vitali Rosati, Valentina Biagioli, Emanuela Tiozzo, Orsola Gawronski, Riccardo Ricci, Antonio Garofalo, Simone Piga, Simone Gramaccioni, Claudio Di Maria, Valentina Vanzi, Alessandra Querciati, Rosaria Alvaro, Luciana Biancalani, Ersilia Buonomo, Mattia Doria, Alberto Villani

**Affiliations:** 1 Health Professionals Development, Continuing Education and Research Service; Bambino Gesù Children’s Hospital, IRCCS, Piazza Sant’Onofrio 4, 00165 Rome, Italy; 2 Italian Pediatric Society, Rome, Italy; 3 Italian Federation of Pediatric Physicians, Rome, Italy; 4 Unit of Clinical Epidemiology, Bambino Gesù Children’s Hospital, IRCCS, Piazza Sant’Onofrio 4, 00165 Rome, Italy; 5 Academic Department of Pediatrics, Bambino Gesù Children’s Hospital, IRCCS, Piazza Sant’Onofrio 4, 00165 Rome, Italy; 6 Department of Biomedicine and Prevention, University of Rome Tor Vergata, Rome, Italy; 7 Pediatric Emergency Department and General Pediatrics, Bambino Gesù Children’s Hospital, IRCCS, Piazza Sant’Onofrio 4, 00165 Rome, Italy

**Keywords:** Community care facilities, Attitude of health personnel, Clinical competence, Health promotion, Pediatric nurses, Pediatricians, Community health nurses

## Abstract

**Background:**

The role played by nurses in caring for children in pediatricians’ officies in the community is crucial to ensure integrated care. In Italy, pediatricians are responsible for the health of children aged 0–14 years living in the community. This study aimed to describe Italian primary care pediatricians’ opinions about the usefulness of several nursing activities that pediatric nurses could perform in pediatricians’ offices.

**Methods:**

An online survey with pediatricians working in primary care in Italy was conducted between April–December 2018. A 40-item questionnaire was used to assess four types of nursing activities: clinical care, healthcare education, disease prevention, and organizational activities. The answers ranged from 1 (not useful at all) to 6 (very useful). Moreover, three open-ended questions completed the questionnaire.

**Results:**

Overall, 707 pediatricians completed the online survey. Participants were mainly female (63%), with a mean age of 57.74 (SD = 6.42). The presence of a pediatric nurse within the pediatrician’s office was considered very useful, especially for healthcare education (Mean 4.90; SD 1.12) and disease prevention (Mean 4.82; SD 1.11). Multivariate analysis confirmed that pediatricians ‘with less working experience’, ‘having their office in a small town’, and ‘collaborating with a secretary and other workers in the office’ rated the nurse’s activities significantly more useful.

**Conclusions:**

A pediatric nurse in the pediatrician’s office can significantly contribute to many activities for children and their families in the community. These activities include clinical care, healthcare education, disease prevention, and the organizational processes of the office. Synergic professional activity between pediatricians and pediatric nurses could ensure higher health care standards in the primary care setting.

**Supplementary Information:**

The online version contains supplementary material available at 10.1186/s12875-021-01457-1.

## Background

Primary care services play a crucial role in providing care to healthy children and adolescents and in coordinating care for pediatric patients who need multidisciplinary support [1, 2]. To strengthen the capacity of the primary pediatric health care model, it is paramount to ensure those resources and services that today continue to be mainly a prerogative of hospitals in several countries [3–5]. In particular, in Italy, a universalistic approach based on a highly-valued community healthcare service has become a concrete reality since the National Healthcare Plan was implemented between 2003 and 2005. One of its objectives is to promote the community as the primary venue for social and healthcare services and health governance. However, considerable efforts are required to ensure integrated and high quality primary care for children throughout the country.

In many countries, like the United Kingdom, Ireland, Portugal, Sweden, and Norway, general practitioners also care for children [6]. In contrast, Italy, like other European countries such as France, Belgium, or Germany, has a combined system where pediatricians care for younger children [7]. In Italy, pediatric primary care is provided by pediatricians, whose services are free of charge for every child from birth until the age of 14 years, and, if affected by severe conditions, also up to the age of 16 years [8, 9]. Pediatricians working in primary care, known as ‘family pediatricians’ in Italy [10–12], play a key role in the prevention, treatment, and rehabilitation of every child/adolescent, as well as providing health education and health promotion, with a focus on children’s physical, mental, relational, and cognitive development [13]. They work according to an arrangement made with the Italian public service through a private-public partnership, which involves solo practices or associative forms such as pediatric group practices, associations, and networks. Their offices are open all day during weekdays [11] and, if necessary, they make house calls. To meet healthcare needs on a 24/7 basis, primary care services are organized as integrated systems, where pediatricians are one of the main pillars.

In recent years, Italian regulations have emphasized the importance for health professionals to provide integrated and multidisciplinary care to children in the community by working together to ensure holistic care also in the primary care setting [14, 15]. An integrated care approach at the community level could improve health outcomes especially for children with chronic conditions and reduce readmission rates [16]. For example, nurses in integrated pediatric primary care services can take part in child health surveillance programs, as also shown by experiences in other countries [17].

While the role of pediatric nurses is well-established within hospital settings, in several countries worldwide more should be done to expand the scope of nursing in the community setting [18–20], where often an overly medical view of primary care prevails [21]. For example, a survey showed that nurses are present in pediatric primary care settings in about 64% of the European countries [6]. Although pediatric nurses play a key role in managing children’s conditions and enhancing symptom- and disease-management skills for the entire family in hospitals [22, 23], they are not always involved in providing this type of care in the community [24, 25]. In adult care, the contribution of family nurses has been increasingly recognized as extremely valuable, cost-effective, and well-accepted [26, 27]. This is desirable also for pediatric primary care, where nurses need to closely collaborate with pediatricians to promote the health of children and their families, especially when dealing with complex cases and treatments [10, 28].

With the support of pediatric nurses, pediatricians could further improve the care of the whole family and facilitate care transitions [29]. The family nursing role in the pediatric context is innovative and could include several activities, such as the assessment of family needs, and planning and coordinating integrated care pathways, while promoting the empowerment of the entire family [30]. In line with the family-centered approach, family nurses establish a partnership of trust with the family to achieve more positive outcomes for children in the community [31]. All the nursing activities concerning disease management should be performed in collaboration with the pediatrician and consistently with the operational procedures and protocols of the pediatrician’s office.

Since the current organization of pediatric primary care in Italy does not mandate the presence of a pediatric nurse in all the pediatricians’ offices, the majority do not have a pediatric nurse [32]. Therefore, it is important to understand how pediatricians could work in close collaboration with pediatric nurses to provide more effective primary care in the community. For example, pediatric nurses could practice collaboratively with pediatricians in their office to foster prevention, education, continuity of care for children with chronic conditions, while reducing inappropriate access to the accident and emergency department [33].

To involve primary care pediatricians in identifying which activities pediatric nurses could perform at their office, a pilot study was previously conducted to describe the pediatricians’ opinions [34]. However, considered the total number of pediatricians in Italy, the sample of those who participated in the previous pilot study was small (*n* = 178) and the questionnaire had so many items that a large number of respondents did not complete it. Therefore, we conducted a similar study, but this time at a national level, to describe the opinions of all the pediatricians working in primary care in Italy regarding the usefulness of several nursing activities that pediatric nurses could perform in their offices.

## Methods

### Design and participants

A cross-sectional study design was used. An online survey was conducted between the end of April and December 2018. Participants were pediatricians working in primary care across Italy who were members of the main Italian pediatric associations or scientific societies. Out of a total of 7656 pediatricians working in primary care in Italy [35], potential participants included 5700 pediatricians registered with the Italian Federation of Pediatricians, the professional association of primary care pediatricians affiliated with the Italian National Health Service [36].

### Ethics

The Ethics Committee of a large academic pediatric research hospital in Italy approved this study. An online consent form was provided in the preliminary section of the survey, including information about the purposes of the study and the data collection process. It was specified that participation in the study was anonymous and voluntary. Individuals who agreed to participate could then click on ‘I agree’ to access the survey.

### Instruments

**Fig. 1:**
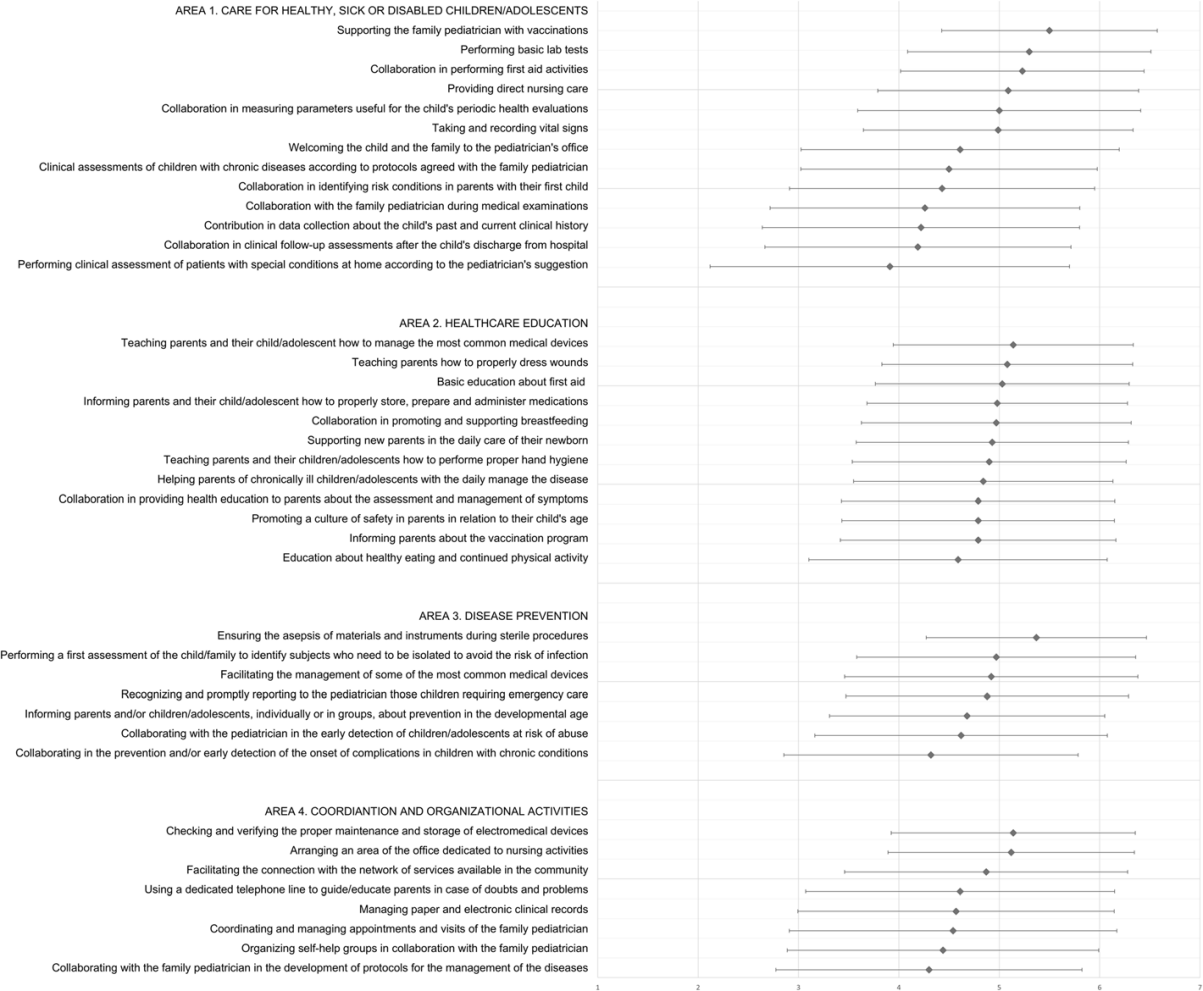
Item scores for each of the 4 Areas

A questionnaire investigating the opinions about the usefulness of nursing activities that could be conducted in the primary care pediatrician’s office was used (Fig. 1). This is the short version of a questionnaire developed from qualitative interviews [34], including 71 items and investigating four areas of nursing activities in the pediatrician’s office. The short version was developed specifically for this study through the steps shown in Supplementary Figure S1. Many stakeholders were involved in this process to ensure that all the different perspectives were taken into account [37]. Overall, the questionnaire included a total of 46 items and 3 open-ended questions. The first 40 + 4 items investigated four areas: Area 1 ‘Care for healthy, sick or disabled children/adolescents’ (13 + 1 items); Area 2 ‘Healthcare education’ (12 + 1 items); Area 3 ‘Disease prevention’ (7 + 1 items); and Area 4 ‘Coordination and organizational activities’ (8 + 1 items). Participants were asked to indicate the extent to which they perceived as useful each nursing activity on a scale from 1 (not useful at all) to 6 (very useful). At the end of each area, respondents had the option to add an extra activity or comment (+ 1). In addition, two items investigated the respondents’ overall opinion about the usefulness of having a pediatric nurse in the pediatrician’s office and whether they would recommend the collaboration of a nurse to a colleague, ranging from 1 (not useful at all) to 6 (very useful). Moreover, three open-ended questions asked participants to add: (1) any extra useful activities to be performed by a nurse in the pediatrician’s office; (2) any other comments; and (3) type of education considered useful for pediatricians and nurses. Items regarding socio-demographic data, type of job, and organizational characteristics were at the end of the survey.

### Data collection

The main Italian Pediatric Association (Italian Federation of Pediatricians) and the Italian Society of Pediatricians invited their members – who were registered as primary care pediatricians – to participate in the study. The link to the survey was sent via e-mail directly to potential participants who were on the mailing lists of these organizations, explaining the purpose of the study and that its results would provide useful information for organizational and educational innovations in the future. A second e-mail was sent to remind the pediatricians to respond to the survey. Moreover, the study was also disseminated through the newsletters of the pediatric associations.

### Data analysis

Descriptive statistics (frequency, percentage, median, interquartile range [IQR], mean, and standard deviation [SD]) were employed to describe the participants’ socio-demographic and job characteristics. Cronbach’s alpha was used to evaluate internal consistency for each of the four areas of nursing activities. The mean (SD) scores for each area were calculated. Associations between scores were investigated using Spearman’s correlation. To identify which area was rated as the most useful one, univariate repeated-measures analysis of variance (ANOVA) was performed using the mean score of each area as a within-subject factor. ANOVA was used to compare the scores of the four areas between respondents completing the entire questionnaire and those who did not provide personal information. The associations between the scores of the four areas and the characteristics of pediatricians were examined through *t-*test or ANOVA, which was conducted using Tukey’s posthoc test. To identify the predictive factors of the scores of each nursing activity area, four multiple linear regression models were developed by using the factors that had univariate *P* values < 0.20 as independent variables. This increased *p*-value cutoff was chosen to account for those independent variables without a significant effect individually but potentially significant when included in a multivariate regression [38]. SPSS Version 22 (Armonk, NY: IBM Corp) was used for statistical analysis.

The qualitative data collected from the open-ended questions were analyzed through inductive content analysis [39].

## Results

### Participant characteristics

The survey was sent to about 5200 pediatricians working in primary care across Italy. Of these, 585 (response rate = 11.3%) participants completed the entire survey including the socio-demographic data (last part of the survey). As shown in Supplementary Figure S2, Tuscany and Lazio were the Regions with the highest numbers of participants.

Participants were mainly female (*n* = 368, 62.9%), their mean age was 57.74 (SD = 6.42), and their median length of work experience as pediatricians in primary care was 25 years (IQR =19–31) (Table 1). They worked in many locations across Italy, slightly more in the north (*n* = 214, 36.6%) and in smaller towns (*n* = 323, 55.2%) with < 20,000 inhabitants (*n* = 183, 31.3%). The office of the primary care pediatrician was often a group practice with other pediatricians (*n* = 211, 36.1%). More than two-thirds (n. 431, 73.7%) employed a secretary, and less than one-third (*n* = 173, 29.6%) employed a nurse.

**Table 1:** Participant characteristics (*n* = 585)

	n	%
Sex		
Male	217	37.1
Female	368	62.9
Age (mean, SD)	57.74	6.42
Age		
≤ 55	150	25.6
56–59	162	27.7
60–62	145	24.8
≥ 63	128	21.9
Work experience (years)		
≤ 10	67	11.5
11–20	100	17.1
21–30	272	46.5
31–40	138	23.6
≥ 41	8	1.4
Workplace		
North	214	36.6
Center	204	34.9
South and Islands	167	28.5
Type of town		
Capital of the Region	107	18.3
Capital of the Province	155	26.5
Other	323	55.2
Size of the town (inhabitants)		
< 20.000	183	31.3
20.000–35.000	73	12.5
35.000–50.000	52	8.9
50.000–100.000	98	16.8
> 100.000	179	30.6
N patients cared		
≤ 850	192	33.4
850–1000	228	39.7
> 1000	155	27.0
N opening days of the office		
5	550	94.7
6	30	5.1
7	1	0.2
Office		
On their own	180	30.8
Associated with pediatricians	211	36.1
Associated with a general practitioner	34	5.8
Group pediatrics	160	27.4
Secretary in the office		
Yes	431	73.7
No	154	26.3
Nurse in the office		
Yes	173	29.6
No	412	70.4
Other workers in the office		
No	469	80.2
Yes (specify)	116	19.8
In-office vaccinations		
Yes	288	49.2
No	297	50.8
Type of vaccinations		
Mandatory	3	1.0
Recommended	123	42.7
Both of them	162	56.3
Vaccination campaigns		
Yes	288	52.5
No	261	47.5
% of work time dedicated to vaccinations		
0%	151	27.4
1–9%	149	27.0
10%	64	11.6
20%	51	9.3
30%	61	11.1
40%	23	4.2
50%	20	3.6
60%	32	5.8
N patients vaccinated per week		
0	222	40.3
1–10	189	34.3
11–20	81	14.7
21–50	52	9.4
> 50	7	1.3
N exempt patients		
0	4	0.7
1–10	114	20.7
11–25	176	31.9
26–50	146	26.5
51–75	49	8.9
76–100	25	4.5
> 100	37	6.7
N patients with disability		
0	6	1.1
1–5	144	26.1
6–10	192	34.8
11–20	124	22.5
> 20	85	15.4

Participants reported that they cared for a median number of 900 patients (IQR = 800–1040). About 80% reported that up to 50 of their patients were exempt from payment because of their particular clinical conditions and that 1 every 20 patients had their disability status recognized. Nearly half of the sample performed in-office vaccinations (*n* = 288, 49.2%), which were often both mandatory and recommended (*n* = 162, 56.3%), and participated in vaccination campaigns (*n* = 289, 52.5%). About 54% reported that vaccination represented less than 10% of all their activities.

### Reliability and scores of the areas

Internal consistency was high: Cronbach’s alpha was 0.92 for Area 1, 0.96 for Area 2, and 0.90 for Areas 3 and 4 (Table 2). Participants significantly rated healthcare education as the most useful area of nursing activity in their office, followed by disease prevention (*p* < .001) (Table 2). The scores of the two items regarding the overall opinion about having a nurse working in the pediatrician’s office were high, indicating that participants had judged positively the presence of a pediatric nurse in the pediatrician’s office (Mean = 5.31, SD = 1.11) and they would recommend the presence of a nurse to a colleague (Mean = 5.30, SD = 1.11). All the scores were positively correlated with one other and with the two items on the overall opinion (*p* < .001). As shown in Supplementary Table S1, participants who completed the whole questionnaire (*n* = 585, 82.7%) reported in every area significantly higher scores than those who did not provide information about their own socio-demographic and work characteristics (*n* = 122, 17.3%), who were thereby excluded from data analyses.

**Table 2:** Mean scores of the four areas and Cronbach’s alpha (*n* = 585)

	Mean (DS)	Cronbach’s alpha
Area 1	4.76 (1.01)	0.92
Area 2	4.94 (1.09)	0.96
Area 3	4.86 (1.07)	0.90
Area 4	4.73 (1.11)	0.90

Note. Area 1: Care for healthy, sick or disabled children/adolescents; Area 2: Healthcare education; Area 3: Disease prevention; Area 4: Coordination and organizational activities

### Scores of the items

In Area 1 (Care for healthy, sick, or disabled children/adolescents), the items with the highest scores were ‘collaboration in vaccinations’, ‘performing minor laboratory tests’, and ‘first aid activities’ (Fig. 1). In Area 2 (Healthcare education), the items with the highest scores were ‘education on medical devices’, ‘correct dressing’, and ‘first aid’. In Area 3 (Disease prevention), the items with the highest scores were ‘ensuring asepsis’, ‘identification of subjects who needed to be isolated’, and ‘facilitating the management of medical devices’. In Area 4 (Coordination and organizational activities), the items with the highest scores were ‘maintenance and storage of electromedical devices’, ‘organizing an area for nursing activities’, and ‘facilitating the connection with the network of services’ (Fig. 1).

### Regression models

After examining univariate associations between the scores of each area and participant characteristics (Supplementary Table S2), we conducted a regression analysis, which confirmed that work experience, type of town, secretary, and other workers in the office were independently associated with Area 1 (Table 3). In particular, those who had less work experience, an office located in a small town, collaborated with a secretary and other workers in the office rated significantly higher the usefulness of a nurse caring for healthy, sick, or disabled children/adolescents in their office (Area 1) compared to others. Regression analysis also confirmed that age, having a secretary and other workers in the office were independently associated with Area 2. In particular, participants who were younger and collaborated with a secretary and other workers in the office rated as more useful the activity of nurses providing healthcare education in their office compared to other participants (Table 3). The independent variables that were significantly associated with the scores of Area 3 were the type of town, having a secretary, and other workers in the office (Table 3). This means that pediatricians who worked in an office located in a small town and collaborated with a secretary and others, rated as more useful the nurse’s activity of disease prevention compared to other participants. Regression analysis also confirmed that work experience, type of town, presence of a nurse in the office, and collaboration with other workers were independently associated with Area 4 (Table 3). In particular, those who had less work experience, an office located in a small town, and collaborated with a nurse and other workers within the office rated significantly higher the usefulness of nurses performing coordination and organizational activities in their office compared to others.

**Table 3:** Regression model predicting the mean scores of the four areas of nursing activity (*n* = 585)

	Area 1		Area 2		Area 3		Area 4	
	β	P	β	P	β	P	β	P
Age	.046	.436	−.087	.039	−.056	.361	.021	.718
Work experience	−.140	.020	–	–	−.088	.152	−.172	.004
Small town	.114	.006	–	–	.118	.005	.094	.023
Secretary in the office	.152	<.001	.138	.001	.133	.002	–	–
Nurse in the office	.060	.145	–	–	–	–	.106	.010
Others in the office	.083	.045	.090	.035	.109	.011	.086	.036
N exempt patients	–	–	.036	.392	–	–	–	–
N disabled patients	–	–	–	–	.086	.072	–	–
*R*^2^	0.053		0.036		0.063		0.049	
F	5.39		5.06		5.23		5.93	
P	<.001		.001		<.001		<.001	

Note: Area 1: Care for healthy, sick or disabled children/adolescents; Area 2: Healthcare education; Area 3: Disease prevention; Area 4: Coordination and organizational activities

### Qualitative findings

Overall, 142 participants out of 585 (24.3%) primary care pediatricians answered to at least one of the open-ended questions (one for each of the 4 Areas and 3 final questions), providing a total of 235 answers. The open-ended question that received the greatest number (*n* = 100; 42.5%) of answers was the one about professional education. Content analysis resulted in 5 main categories and 19 sub-categories (Table 4). Participants highlighted many positive aspects regarding the nurse’s activities in the pediatrician’s office (*n* = 156; 66.4%) such as clinical assessment (triage) and professional integration. Moreover, few negative aspects (*n* = 13; 5.5%) emerged from the participants’ responses, such as the issue of the professional scope of practice. A considerable number of critical considerations (*n* = 66; 28.1%) also emerged from participants. Most of these regarded training on the field with an experienced pediatrician, transition from individual work to teamwork and, sustainability of the professional role considering trade union or contractual issues.

**Table 4:** Qualitative findings

Categories	Sub-categories	***Verbatim data extracts***
Clinical assessment of the child/adolescent	Health assessment	*Speak to the parents before performing the child’s periodic health assessment*
	Anamnesis and triage	*Adequately trained nurses are […*] *on the lookout for risky situations […] that often very busy pediatricians may underestimate*
	Specific diagnostic tests	*Perform some diagnostic tests that require specific professional competencies (electrocardiogram, spirometry, prick test)*
Patient and/or parent education	Health promotion	*Collaborate with pediatricians in providing health education, such as correct lifestyle habits*
	Relationship with child/adolescent and family	*Listening to adolescents without parents, to try to develop an empathic relationship and encourage dialogue*
	Web use	*Recommend [the best] science websites*
Professional integration	Team value	*Nursing staff constitutes a unique and indispensable added value*
	Professional borders	*Recognize professional autonomy as synergistic to that of the pediatrician, but each with their own specificities*
	Improving the quality of care	*Organizational changes and the sharing of clinical-healthcare activities have enabled to significantly improve the quality of care*
	Useful only to assist with vaccinations	*Nurses are useful only when pediatricians administer vaccines*
Sustainability	Unsustainable costs	*We had to give up despite the precious help, because the costs incurred were no longer sustainable*
	Medical and legal issues	*More procedures regarding safety at work are needed: more hours of training, occupational medicine examinations, extra costs for the employer*
	Dedicated spaces for nursing activities	*The main problem I notice is finding a dedicated space for nurses to perform their activities.*
	Secretarial support	*I have had an office assistant for 18 years. She is not a nurse but a secretary with nursing skills who helps me with total commitment.*
Professional education	Graduate or post-graduate university degree.	*Specific pediatric specialization after the nursing bachelor’s degree, maybe with an evidence-based nursing course and a mandatory internship in the pediatrician’s office.*
	Clinical placements	*It would be useful to arrange placements for nursing students at the pediatrician’s office.*
	Specific continuing education	*Conferences and educational sessions dedicated to specific and general topics focusing on the collaboration between pediatricians, nurses and families*
	Shared education	*Specific nature of the activities performed in the pediatrician’s office, which requires collaborative training*
	Clinical assessment/ counselling / organizational topics	*Counselling, parenting support, telephone triage, screening, vaccinations, psychomotor development of the child*

## Discussion

This study investigated the opinions of Italian primary care pediatricians about the usefulness of several nursing activities that pediatric nurses could perform in their offices. In line with the pilot study [34], participants had a positive opinion of having a pediatric nurse in their office and rated ‘very useful’ most of the suggested activities. This is promising in light of the crucial role that pediatric nurses could play in the community setting not only in Italy [40] but in any other country [41, 42].

‘Healthcare education’ was the area of nursing practice that was rated as the most useful one, in line with the pilot study [34]. This may reflect the great importance given to patient and family education in the community setting [43, 44] and the high consideration for nurses’ educational competencies worldwide [45, 46]. Pediatric nurses could perform many educational activities in the community to support the pediatricians in empowering children/adolescents and their parents in terms of health promotion, risk-prevention, disease management, and improved adherence to treatment. For example, education about how to manage medical devices was rated as the most useful one, in line with the needs of patients with chronic diseases [47–49]. Moreover, the pediatrician’s office is one of the main settings in the community where parents of healthy children refer to [9]. Therefore, nurses could play an important role in promoting healthy lifestyles in this setting [10] and also contribute to the system’s effort to create multidisciplinary teams to promote holistic health for children [50].

The second area rated as the most useful one was ‘Disease prevention’, whereas in the pilot study this was ‘Care for healthy, sick or disabled children/adolescents’ [34]. This may be due to pediatricians’ increased awareness of the role nurses play in disease prevention, such as vaccination. Although not every pediatrician provides mandatory and recommended vaccinations based on regional agreements, nurses’ collaboration in vaccination was rated as one of the most important activities. Nurses’ immunization activities under the responsibility of primary care pediatricians include cooperation in advocating for mandatory vaccines [51], storage of the medication, parent and child education about the procedure, and performing the vaccination. In particular, those who could administer vaccines rated the role of nurses as more useful for this crucial service, which often requires teamwork to be widely performed [52, 53]. Operational support for the pediatrician and the educational role for families about crucial aspects of vaccination, such as the complex issue of vaccine hesitancy, may explain this finding [54]. Besides, only a few Italian Regions already had local agreements that supported collaboration with a pediatric nurse in the primary care pediatrician’s office. This may be due to the difficult sustainability of a pediatric nurse in their office, which was expressed by some participants in the qualitative findings. In the future, the issue of providing major support to implement collaboration with nurses in the pediatrician’s office would deserve further discussion.

Compared to the pilot study [34], participants considered less useful the area ‘Care for healthy, sick or disabled children/adolescents’, and, in particular, the nurse’s role in caring for sick children at home was rated as secondary. In Italy, it is possible that in the future, with the support of appropriate organizational processes and instruments (e.g. protocols, e-health, measurement scales), pediatric nurses working in primary care will play a major role in caring for children at home [55]. Also area 4 ‘Coordination and organizational activities’ was considered secondary, as it obtained the lowest score, while area 3 ‘Disease prevention’ obtained the lowest score in the pilot study [34]. This may indicate that participants recognized the uniqueness of nursing competence and training rather than considering them only for assistance with their medical itinerary or with administrative issues. However, some organizational activities were considered to be quite useful, especially by those who already had a nurse in their office and appreciated the support from a health professional that mainly performs healthcare, educational, and prevention activities. With regard to collaboration, those who already had a secretary in their office rated nursing clinical, educational, and prevention activities as more useful. We could assume that the experience of collaborating with others in the primary care office may facilitate collaboration with nurses and foster a better opinion about nursing activities. Therefore, implementing and maintaining a good pediatrician-nurse collaboration is key to providing high-quality comprehensive care and to reciprocally appreciate each other’s professional value [56].

The regression analysis showed other interesting associations. On the one hand, the younger pediatricians had a better opinion about the importance of conducting educational activities. Therefore, younger pediatricians may be more willing to collaborate with nurses in educational activities, given the increasing importance of education for patient care and the potential of nurses in this key role [44, 57]. On the other hand, a longer working experience predicted a poorer opinion of the nursing clinical and organizational activities. This may be the result of their adjustment to being used to working alone for many years. In addition, working in small towns was found to predict a better opinion of nurses’ clinical, educational, and organizational activities. Probably in small towns pediatricians become even more important for patients in the community, because in these places it is often more difficult to reach hospitals or other health services. Therefore, they may value more the need to collaborate with a pediatric nurse in their office.

Overall, qualitative findings showed that pediatricians recognize the need for the specific professional education of pediatric nurses in providing nursing care to children in their office. This type of education should be provided both academically and through continuing education. Shared education and clinical placements were suggested, as pediatricians were interested in working together with pediatric nurses who are well prepared to work in their office. Nursing knowledge and theory development should be better linked to practice-relevant actions [58] so that pediatric nurses develop a specialized understanding of the needs of sick children and their families [59]. This should be taken into consideration to inform undergraduate and postgraduate education curricula for pediatric nurses.

### Limitations

The findings of this study should be interpreted in light of few limitations. Given that there are a total of 7656 pediatricians working in primary care in Italy [35], 9.2% of them participated in this study (12.4% of potential participants, who were the 5700 pediatricians registered with the Italian Federation of Pediatricians [36]). Thereby, the sample cannot be considered representative of the entire population of pediatricians working in primary care in Italy. Moreover, self-selection bias may have occurred [60], as differences in the scores between those who completed and those who did not complete the entire survey were significant. This might indicate that participants with a better opinion of the nursing activities in the pediatrician’s office may have selected themselves by completing the whole survey [61, 62]. In addition, we did not investigate what knowledge and understanding the pediatricians had of the competencies, skills, and responsibilities of pediatric nurses. This may have affected the validity of the results. Future studies should also investigate the opinions of pediatric nurses as well as parents about the need for nursing activities in primary health care. Another limitation is that primary care in Italy is connected to the National Healthcare System, but the main providers are the Regional services, with several differences across Regions. We also recognize that the peculiarity of the Italian health care system limits the generalizability of the findings.

## Conclusions

Overall, a pediatric nurse in the pediatrician’s office can significantly contribute to many activities for children and their families in the community. These activities include clinical care, healthcare education, disease prevention, and the organizational processes of the office. In particular, education and prevention could be the main activities of this new nursing role, which needs to be supported in undergraduate programs and through specific continuing professional education. The activities of pediatric nurses in pediatricians’ offices could support and integrate the important role played by pediatricians in primary care. Further knowledge is needed to secure interprofessional collaboration between pediatric nurses and pediatricians in primary care. In this way, the synergic professional activities between pediatricians and pediatric nurses could ensure higher health care standards in the primary care setting.

## Supplementary Information

**Additional file 1: Table S1.** Mean scores of the four areas and comparison between groups.

**Additional file 2: Table S2.** Comparisons of the scores of the four areas by participants’ characteristics.

**Additional file 3: Figure S1.** Flow chart of the questionnaire reduction process from 71 to 40 items.

**Additional file 4: Figure S2.** The distribution of pediatricians working in primary care across the Italian Regions (*n* = 585).

## Data Availability

The datasets used during the current study are available from the corresponding author on reasonable request. The English language version of the questionnaire developed for this study is available from the corresponding author.

## Abbreviations

IQR: Interquartile range
SD: Standard deviation
