# Supplementary material for: Pediatric nurses in pediatricians’ offices: a survey for primary care pediatricians
Source: BMC Fam Pract. 2021 Jun 29;22:136. doi: 10.1186/s12875-021-01457-1 (PMC8243477; doi:10.1186/s12875-021-01457-1)
Supplement: Supplementary file 1 — Additional file 1: Table S1. Mean scores of the four areas and comparison between groups. [file 12875_2021_1457_MOESM1_ESM.docx]

**Supplementary materials**

**Table S1.** Mean scores of the four areas and comparison between groups

|  | Total sample  (n = 707)  Mean (DS) | Completed the survey  (n = 585)  Mean (DS) | Not completed the survey  (n = 122)  Mean (DS) | P |
| --- | --- | --- | --- | --- |
| Area 1 | 4.71 (1.05) | 4.76 (1.01) | 4.45 (1.21) | .003 |
| Area 2 | 4.90 (1.12) | 4.94 (1.09) | 4.64 (1.27) | .016 |
| Area 3 | 4.82 (1.11) | 4.86 (1.07) | 4.47 (1.38) | .006 |
| Area 4 | 4.70 (1.14) | 4.73 (1.11) | 4.36 (1.41) | .023 |

*Note. Area 1: Care for healthy, sick or disabled children/adolescents; Area 2: Healthcare education; Area 3: Disease prevention; Area 4: Coordination and organizational activities.*
