# Supplementary material for: Pediatric nurses in pediatricians’ offices: a survey for primary care pediatricians
Source: BMC Fam Pract. 2021 Jun 29;22:136. doi: 10.1186/s12875-021-01457-1 (PMC8243477; doi:10.1186/s12875-021-01457-1)
Supplement: Supplementary file 2 — Additional file 2: Table S2. Comparisons of the scores of the four areas by participants’ characteristics. [file 12875_2021_1457_MOESM2_ESM.docx]

**Table S2**. Comparisons of the scores of the four areas by participants’ characteristics

|  | Area 1  Mean (SD) | P |  | Area 2  Mean (SD) | P |  | Area 3  Mean (SD) | P |  | Area 4  Mean (SD) | P |
| --- | --- | --- | --- | --- | --- | --- | --- | --- | --- | --- | --- |
| Sex  Male  Female | 4.79 (1.01)  4.75 (1.00) | .618 |  | 4.95 (1.10)  4.94 (1.09) | .944 |  | 4.89 (1.07)  4.85 (1.08) | .704 |  | 4.77 (1.06)  4.70 (1.14) | .484 |
| Age  ≤ 55  56-59  60-62  ≥ 63 | 4.79 (1.03)  4.90 (0.90)  4.65 (1.08)  4.69 (1.00) | .121 |  | 5.01^ab^ (1.03)  5.12^a^ (1.00)  4.79^b^ (1.17)  4.82^ab^ (1.14) | .022 |  | 4.97^ab^ (1.05)  5.05^a^ (0.93)  4.67^b^ (1.16)  4.72^b^ (1.11) | .003 |  | 4.87^a^ (1.17)  4.91^a^ (0.96)  4.46^b^ (1.12)  4.63^ab^ (1.16) | .001 |
| Work experience  ≤ 10  11-20  21-30  > 30 | 4.96 (1.10)  4.88 (0.85)  4.72 (1.01)  4.68 (1.04) | .139 |  | 5.11 (0.14)  5.07 (0.85)  4.89 (1.10)  4.88 (1.17) | .248 |  | 5.09 (1.15)  5.00 (0.91)  4.83 (1.05)  4.73 (1.15) | .064 |  | 5.06^a^ (1.21)  4.92^ab^ (0.91)  4.63^b^ (1.12)  4.63^b^ (1.13) | .006 |
| Workplace  North  Center  South and Islands | 4.75 (0.98)  4.78 (0.97)  4.76 (1.08) | .954 |  | 4.95 (1.07)  4.88 (1.11)  5.01 (1.09) | .506 |  | 4.84 (1.05)  4.86 (1.09)  4.90 (1.09) | .873 |  | 4.76 (1.10)  4.67 (1.09)  4.76 (1.16) | .607 |
| Type of the town  Capital of the region  Capital of the province  Other | 4.53^b^ (1.12)  4.74^ab^ (1.10)  4.85^a^ (0.90) | .014 |  | 4.80 (1.23)  4.92 (1.22)  5.00 (0.97) | .231 |  | 4.69^b^ (1.25)  4.78^ab^ (1.22)  4.96^a^ (0.92) | .037 |  | 4.49^b^ (1.28)  4.68^ab^ (1.21)  4.83^a^ (0.98) | .022 |
| Size of the town  < 20.000  20.000-35.000  35.000-50.000  50.000-100.000  > 100.000 | 4.80 (0.93)  4.91 (0.94)  4.76 (0.91)  4.81 (1.07)  4.65 (1.09) | .350 |  | 4.89 (1.03)  5.14 (0.94)  5.02 (0.88)  4.97 (1.20)  4.88 (1.19) | .427 |  | 4.86 (0.98)  5.11 (0.96)  4.93 (0.96)  4.86 (1.16)  4.75 (1.17) | .198 |  | 4.78 (0.99)  5.01 (0.98)  4.76 (1.00)  4.69 (1.20)  4.57 (1.24) | .060 |
| N patients cared  ≤ 850  850-1000  > 1000 | 4.76 (1.06)  4.71 (1.09)  4.85 (0.80) | .439 |  | 4.93 (1.12)  4.89 (1.17)  5.01 (0.91) | .608 |  | 4.83 (1.15)  4.85 (1.13)  4.92 (0.87) | .690 |  | 4.75 (1.14)  4.66 (1.18)  4.79 (0.97) | .463 |
| N opening days of the office  5  6-7 | 4.77 (0.99)  4.85 (0.98) | .675 |  | 4.95 (1.08)  5.00 (0.85) | .804 |  | 4.87 (1.07)  4.90 (0.86) | .884 |  | 4.74 (1.10)  4.82 (1.06) | .666 |
| Office  Single  Associated with pediatricians  Associated with family doctors  Group pediatrics | 4.67 (1.06)  4.79 (0.99)  4.68 (1.27)  4.86 (0.89) | .359 |  | 4.84 (1.14)  4.96 (1.11)  4.89 (1.15)  5.05 (0.99) | .349 |  | 4.81 (1.10)  4.86 (1.08)  4.71 (1.29)  4.96 (0.98) | .481 |  | 4.71 (1.14)  4.72 (1.14)  4.80 (1.19)  4.75 (1.02) | .972 |
| Secretary in the office  Yes  No | 4.85 (0.95)  4.53 (1.13) | .001 |  | 5.03 (1.01)  4.69 (1.26) | .001 |  | 4.94 (1.01)  4.65 (1.22) | .004 |  | 4.75 (1.08)  4.67 (1.20) | .429 |
| Nurse in the office  Yes  No | 4.87 (0.89)  4.72 (1.05) | .098 |  | 4.99 (1.07)  4.92 (1.10) | .494 |  | 4.90 (1.02)  4.85 (1.09) | .619 |  | 4.89 (0.99)  4.66 (1.15) | .023 |
| Other workers in the office  No  Yes (specify) | 4.72 (1.04)  4.94 (0.84) | .039 |  | 4.89 (1.14)  5.15 (0.84) | .021 |  | 4.81 (1.11)  5.07 (0.89) | .022 |  | 4.69 (1.13)  4.89 (1.03) | .083 |
| In-office vaccinations  Yes  No | 4.82 (0.97)  4.71 (1.04) | .208 |  | 4.92 (1.09)  4.97 (1.08) | .606 |  | 4.88 (1.06)  4.85 (1.08) | .765 |  | 4.77 (1.11)  4.69 (1.12) | .420 |
| Type of vaccinations  Mandatory/recommended  Both types | 4.77 (1.01)  4.84 (0.96) | .541 |  | 4.99 (1.06)  4.86 (1.13) | .292 |  | 4.92 (1.06)  4.84 (1.08) | .535 |  | 4.82 (1.13)  4.72 (1.11) | .453 |
| Vaccination campaigns  Yes  No | 4.78 (1.00)  4.77 (1.00) | .978 |  | 4.90 (1.06)  4.98 (1.11) | .401 |  | 4.87 (1.06)  4.85 (1.09) | .840 |  | 4.74 (1.08)  4.72 (1.14) | .836 |
| Work proportion for vaccinations  0%  1-10%  10-20%  30-60% | 4.77 (0.97)  4.76 (1.03)  4.80 (0.89)  4.78 (1.11) | .989 |  | 5.06 (0.95)  4.90 (1.14)  4.92 (0.96)  4.85 (1.26) | .394 |  | 4.92 (0.99)  4.75 (1.14)  4.88 (0.95)  4.90 (1.17) | .543 |  | 4.75 (1.06)  4.65 (1.20)  4.79 (1.01)  4.75 (1.14) | .759 |
| N patients vaccinated per week  0  1-10  11-20  > 20 | 4.76 (0.99)  4.77 (1.04)  4.89 (0.87)  4.73 (1.10) | .760 |  | 5.03 (1.02)  4.90 (1.12)  4.92 (1.08)  4.71 (1.19) | .239 |  | 4.89 (1.05)  4.82 (1.10)  4.92 (1.01)  4.78 (1.15) | .787 |  | 4.72 (1.10)  4.73 (1.17)  4.79 (0.96)  4.71 (1.12) | .970 |
| N patients with exemption  0-10  11-25  26-50  51-75  >75 | 4.83 (098)  4.75 (0.95)  4.76 (0.92)  4.95 (1.03)  4.64 (1.31) | .558 |  | 4.79 (1.07)  4.92 (1.12)  5.02 (0.96)  5.27 (0.89)  4.77 (1.38) | .059 |  | 4.79 (1.09)  4.86 (1.07)  4.89 (0.97)  5.04 (1.00)  4.78 (1.31) | .678 |  | 4.73 (1.06)  4.76 (1.08)  4.67 (1.05)  4.96 (1.11)  4.62 (1.39) | .515 |
| N patients with handicap  0-5  6-10  >10 | 4.75 (1.08)  4.74 (0.98)  4.83 (0.97) | .614 |  | 4.86 (1.20)  4.90 (1.05)  5.02 (1.03) | .346 |  | 4.74 (1.17)  4.85 (1.05)  4.95 (1.00) | .179 |  | 4.74 (1.11)  4.71 (1.11)  4.75 (1.11) | .928 |

*Note.* Area 1: Care for healthy, sick or disabled children/adolescents; Area 2: Healthcare education; Area 3: Disease prevention; Area 4: Coordination and organizational activities. P: signiﬁcant difference between means (*t*-test/ANOVA). Means by column with different superscript letters (^a^higher mean; ^b^lower mean) are signiﬁcantly different from each other at pair comparisons with Tukey post hoc test.
