# Supplementary figures and images for: Pediatric nurses in pediatricians’ offices: a survey for primary care pediatricians
Source: BMC Fam Pract. 2021 Jun 29;22:136. doi: 10.1186/s12875-021-01457-1 (PMC8243477; doi:10.1186/s12875-021-01457-1)

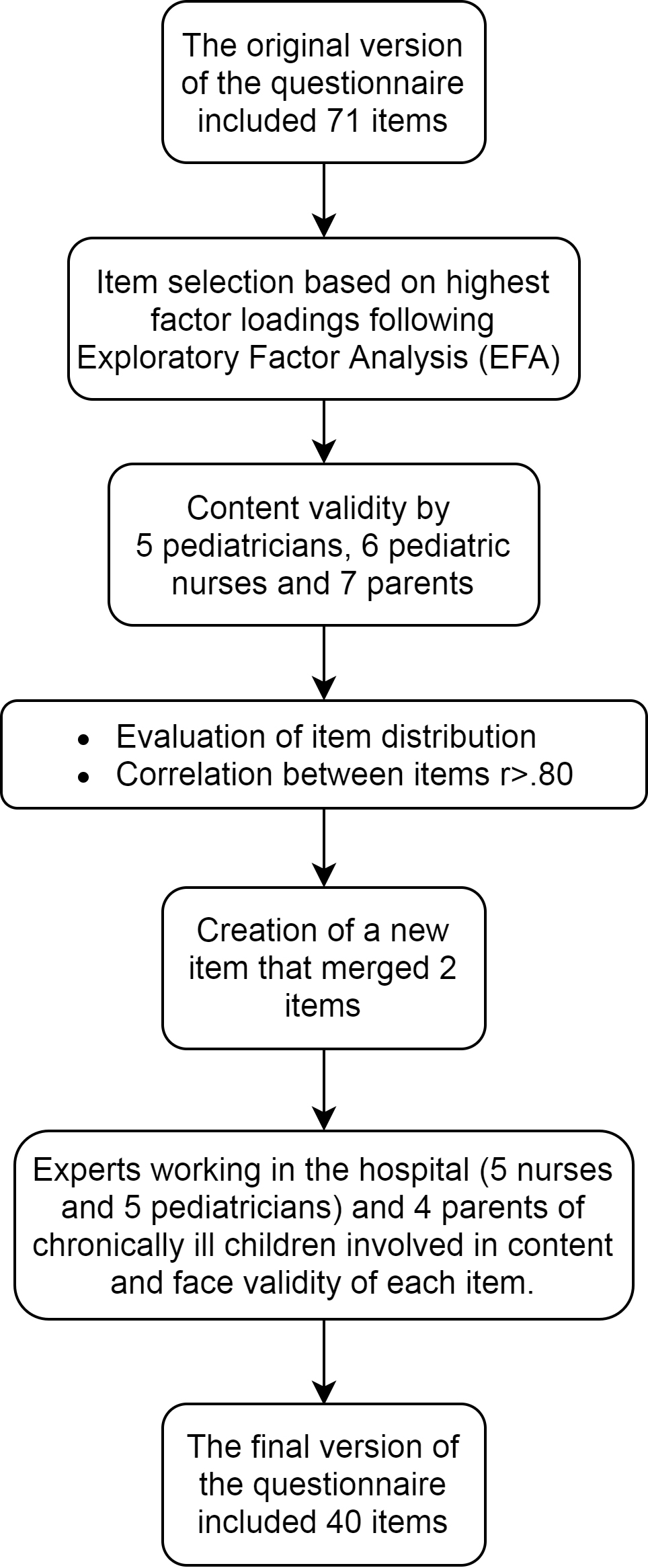

Supplement: Supplementary file 3 — Additional file 3: Figure S1. Flow chart of the questionnaire reduction process from 71 to 40 items. [file 12875_2021_1457_MOESM3_ESM.jpg]

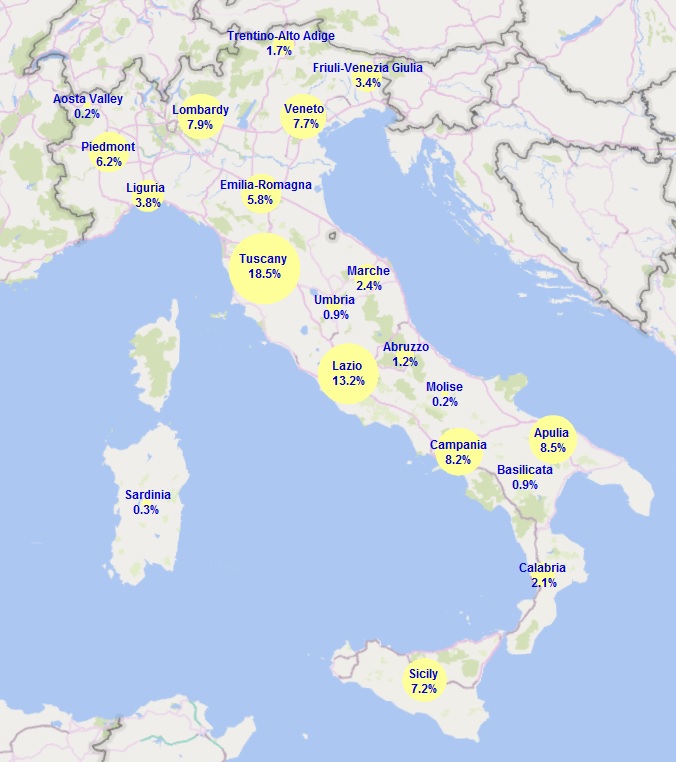

Supplement: Supplementary file 4 — Additional file 4: Figure S2. The distribution of pediatricians working in primary care across the Italian Regions (n = 585). [file 12875_2021_1457_MOESM4_ESM.jpg]
